# Sacrococcygeal Teratoma with Yolk Sac Component in a Neonate

**Published:** 2015-07-01

**Authors:** Ashwini Chandrasekhar Khanolkar, Nirali Chirag Thakkar, Yogesh Kumar Sarin

**Affiliations:** Department of Pediatric Surgery, Maulana Azad Medical College, New Delhi, India

**Keywords:** Sacrococcygeal teratoma, Yolk sac tumor, Neonate

## Abstract

Sacrococcygeal teratoma (SCT) is the most common congenital tumor in neonates. SCT may be benign (mature and immature) or malignant. Malignancy is rare in SCT diagnosed before 2 months of age. Here, we describe a neonate having a mature teratoma with a focus of malignant yolk sac tumor within and the difficulties faced in its management.

## INTRODUCTION

SCT may be benign (mature and immature) or malignant. Malignancy is very rare in infants less than two months of age (7% in boys and 10% in girls) [1].Yolk sac tumors (YST) are rare and highly malignant tumors, occurring in children as well as in young adults. Although most germ cell tumors in children originate in the gonads, the most common primary site for YST is the sacrococcygeal region [2]. Here we describe a rare case of a mature teratoma with a focus of malignant yolk sac tumor within in a neonate and the difficulties faced in its management.

## CASE REPORT

A 2.2kg baby girl, born of a full-term vaginal delivery, presented to us on day 1 of life with a sacrococcygeal swelling. The neonate had passed urine and meconium and was breast feeding well. Local examination revealed 5 x 8 cm cystic, lobulated, trans-illuminant swelling extending up to anus, with the tip of coccyx protruding outwards [Figure.1]. There was no evidence of any neurological deficit.

**Figure:**
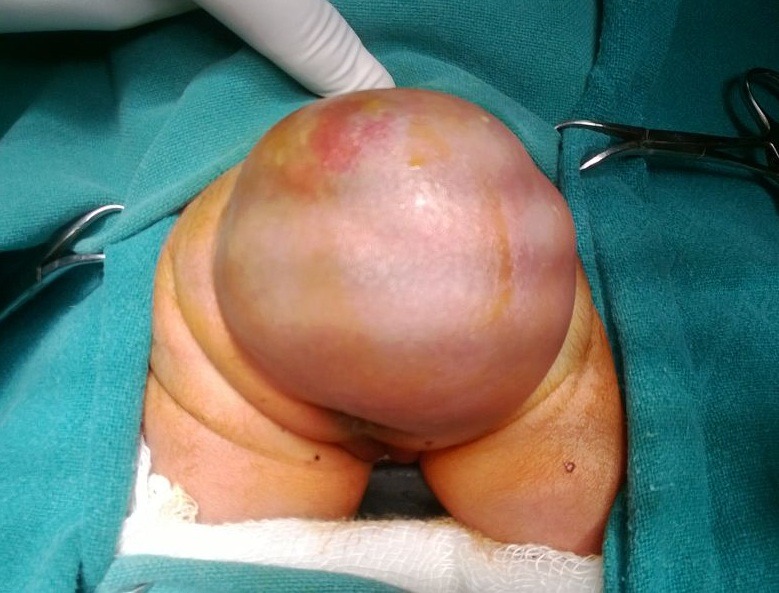
Figure 1: Sacrococcygeal swelling

Antenatal ultrasounds in mother were unable to diagnose the tumor. Ultrasound of the sacrococcygeal region in baby showed evidence of a cystic lesion of 5.9 x 2.7cm size with spina bifida in L5-S1. Her alfa-feto protein level (40606ng/mL) were in the normal range for her age group (normal range for new born- 48406+/-34718ng/mL) [4]. Non-contrast computed tomography (CT) scan of pelvis revealed a large hypodense cystic lesion in the subcutaneous tissue of lower back in close approximation with the lower sacral vertebra with evidence of dural ectasia [Figure 2].

**Figure:**
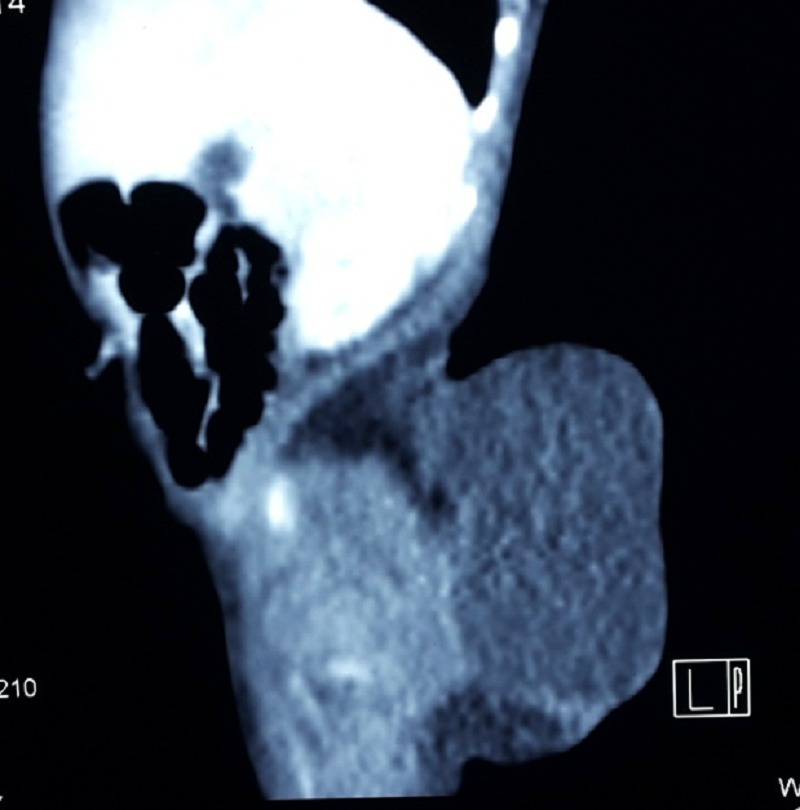
Figure 2: CT picture

Intra-operatively, a 10x8x8 cm cystic swelling with a 0.5x0.5cm solid nodular component within was seen. There was no intra-pelvic extension. Surgical excision of the SCT with coccygectomy was done. Histopathological report revealed a mature teratoma with a small focus of yolk sac component measuring 0.5cm diameter, surrounded by a thin rim of fibrous tissue. Her metastatic work up was done, which included CT chest (normal study). AFP and beta Human Chorionic Gonadotropin levels after surgery were 1947ng/ml and less than 0.1mIU/ml (both normal for age).

There was no consensus on the management in the tumor board; the pediatric oncologists favored administration of adjunct chemotherapy, whereas few pediatric surgeons and histopathologists favored ‘wait and watch’ policy. We opted in favor of chemotherapy as our patient came from a far distance and close surveillance was difficult. Before starting chemotherapy, her baseline hemogram, kidney and liver function were done. The baby was given three cycles of chemotherapy. After the first cycle of chemotherapy, the baby developed diarrhea, mucositis, electrolyte disturbances, febrile neutropenia and severe thrombocytopenia, for which appropriate therapy was given. The subsequent cycles she tolerated well with no side – effects. At 6 months follow-up, she is doing well.

## DISCUSSION

The imaging of sacral tumors in children is valuable to look for foci of malignancy. YST, consisting of fat-free soft tissue, is often complicated with hemorrhage, necrosis and cystic degeneration. Honeycomb-like change is an imaging characteristic of YST [4]. Loss of planes between tumor and surrounding tissue, sacral invasion and metastases are suggestive of malignancy. YST element was just a small focus in our case; therefore, such changes were not seen.

AFP is a useful tumor marker for diagnosis and monitoring of therapy. However, AFP levels are normally increased in neonates and reach adult values by 8 months of age. Thus normal AFP levels for age should be taken into consideration while interpreting the results [3].

The management of SCT is mainly surgical with complete excision of the tumor. The treatment of malignant SCTs, such as primary yolk sac tumor is dependent on the extent of disease. Local disease is best managed surgically, while advanced tumor stages benefit best from neoadjuvant platinum based chemotherapy followed by surgery. Survival rates using these strategies are higher than 80% [5].

As per NCI guidelines, surgery and chemotherapy with four to six cycles of standard cisplatin, etoposide, and bleomycin (PEB) gives an overall survival (OS) outcome greater than 90% [6]. However, recent studies show that the JEB regime (carboplatin, etoposide and bleomycin) gives a superior response as compared to PEB in the treatment of malignant SCT [7]; it is to be given for 4 courses or to be continued for 2 courses beyond documented Complete Response. Bleomycin is to be omitted in children less than 1 year age as per the UKCCSG GCII guidelines [8]. 

Another school of thought is to keep a regular follow up of patients having complete excision of the malignant focus with clear resection margins. In such cases, chemotherapy is only begun if there is evidence of recurrence. The rationale for this approach is to avoid cytotoxic chemotherapy in the neonatal range. However, the recurrence rate of malignant SCT is 33 percent [1]. Also, a CCG-POG study reported that four of six children with malignant recurrence after neonatal resection of mature or immature teratomas were noted to have microscopic foci of YST in the original specimen [9]. In the developing countries, where majority of the patients belong to poor families with low socioeconomic status and come from remote and far off places, it is very difficult for them to adhere to the strict and regular follow up required in such cases. As there is a high risk that these patients may be lost to follow up and present later with a large recurrence, we prefer to give four cycles of upfront chemotherapy (JEB regime) to our patient.

## Footnotes

**Source of Support:** Nil

**Conflict of Interest:** The author is editor of the journal but he is not involved in decision making of the manuscript.

